# 4,4′-Bipyridine-1,1′-diium 2,3,5,6-tetra­bromo­terephthalate dihydrate

**DOI:** 10.1107/S1600536811035926

**Published:** 2011-09-14

**Authors:** Hitoshi Kumagai, Satoshi Kawata

**Affiliations:** a Institute for Molecular Science, 38 Nishigounaka Okazaki, Aichi, Japan; b Department of Chemistry, Fukuoka University, Fukuoka 814-0180, Japan

## Abstract

The title compound, C_10_H_10_N_2_^2+^·C_8_Br_4_O_4_^2−^·2H_2_O, consists of a tetra­bromo­terephthalate dianion, a 4,4′-bipyridinium dication and two solvent water mol­ecules. Crystallographic inversion centers are situated at the center of the aromatic ring of the dianion as well as at the midpoint of the carbon–carbon bond connecting the pyridine rings in the dication. In the crystal, inter­molecular N—H⋯O hydrogen-bonding inter­actions between tetra­bromo­terephthalate dianions and protonated 4,4′-bipyridinium dications result in the formation of a chain-like structure. Further O—H⋯O hydrogen bonds between carboxyl­ate O atoms and water mol­ecules lead to the formation of a two-dimensional network in the crystal structure.

## Related literature

For hydrogen-bonded assemblies, see: Desiraju & Steiner (1999 ▶); Jia *et al.* (2009 ▶); Soleimannejad *et al.* (2009 ▶). For proton transfer, see: Kawata *et al.* (2002 ▶).
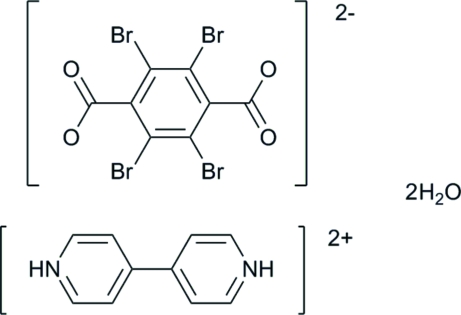


## Experimental

### 

#### Crystal data

C_10_H_10_N_2_^2+^·C_8_Br_4_O_4_^2−^·2H_2_O
*M* *_r_* = 673.93
Triclinic, 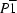

*a* = 6.503 (3) Å
*b* = 9.249 (4) Å
*c* = 9.987 (4) Å
α = 64.119 (14)°
β = 85.868 (18)°
γ = 73.737 (14)°
*V* = 517.9 (4) Å^3^
*Z* = 1
Mo *K*α radiation
μ = 7.83 mm^−1^
*T* = 293 K
0.30 × 0.20 × 0.20 mm

#### Data collection

Rigaku Mercury70 diffractometer
Absorption correction: multi-scan (*REQAB*; Rigaku, 1998 ▶) *T*_min_ = 0.126, *T*_max_ = 0.209
5065 measured reflections
2336 independent reflections
2116 reflections with *F* ^2^ > 2.0σ(*F* ^2^)
*R*_int_ = 0.027

#### Refinement

*R*[*F* ^2^ > 2σ(*F* ^2^)] = 0.031
*wR*(*F* ^2^) = 0.086
*S* = 1.19
2336 reflections
136 parameters
H-atom parameters constrained
Δρ_max_ = 0.48 e Å^−3^
Δρ_min_ = −1.55 e Å^−3^

### 

Data collection: *CrystalClear* (Rigaku/MSC, 2005 ▶); cell refinement: *CrystalClear*; data reduction: *CrystalClear*; program(s) used to solve structure: *IL MILIONE* (Burla *et al.*, 2007 ▶); program(s) used to refine structure: *SHELXL97* (Sheldrick, 2008 ▶); molecular graphics: *CrystalMaker* (Palmer, 2004 ▶); software used to prepare material for publication: *CrystalStructure* (Rigaku, 2010 ▶).

## Supplementary Material

Crystal structure: contains datablock(s) global, I. DOI: 10.1107/S1600536811035926/im2306sup1.cif

Structure factors: contains datablock(s) I. DOI: 10.1107/S1600536811035926/im2306Isup2.hkl

Supplementary material file. DOI: 10.1107/S1600536811035926/im2306Isup3.cdx

Supplementary material file. DOI: 10.1107/S1600536811035926/im2306Isup4.cml

Additional supplementary materials:  crystallographic information; 3D view; checkCIF report

## Figures and Tables

**Table 1:** Hydrogen-bond geometry (Å, °)

*D*—H⋯*A*	*D*—H	H⋯*A*	*D*⋯*A*	*D*—H⋯*A*
O3—H6⋯O2^i^	0.963	1.944	2.822 (4)	150.5
O3—H7⋯O2	1.002	1.765	2.766 (3)	177.2
N1—H5⋯O1^ii^	0.941	1.670	2.606 (4)	172.9

Symmetry codes: (i) 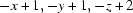; (ii) 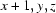.

**Fig. 1.**
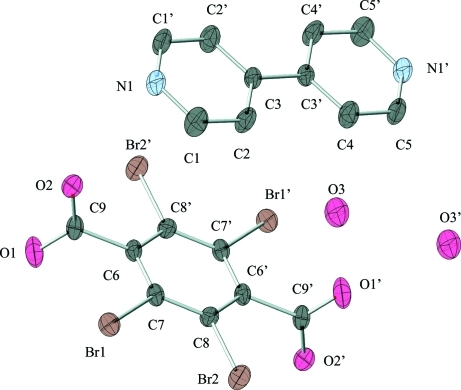
ORTEP drawing of the title compound showing 50% probability displacement ellipsoids.

**Fig. 2.**
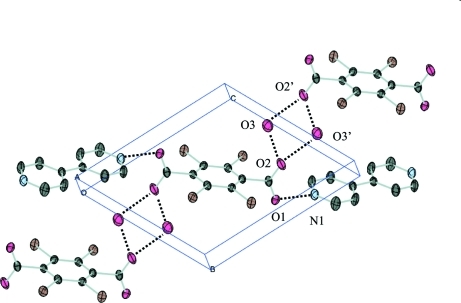
Hydrogen bonding interactions for the title compound.

## supplementary crystallographic information

### Comment

Hydrogen bonded inorganic and organic compounds are of great current interest in recent years due to fundamental scientific and technological applications (Desiraju & Steiner,1999; Kawata *et al.*, 2002). Here we report the synthesis and single-crystal structure of the title compound [(C_8_Br_4_O_4_)(C_10_H_10_N_2_).2H_2_O)]. It consists of a tetrabromoterephthalate dianion, a 4,4'-bipyridinium dication and solvent water molecules. Intermolecular N–H···O hydrogen bonding interactions between tetrabromoterephthalate dianions and protonated 4,4'-bipyridinium dications result in the formation of a one-dimensional chain-like structure. Further O–H···O hydrogen bonds between oxygen atoms of carboxylates and water molecules lead to the formation of a two-dimensional network in the crystal structure.

### Experimental

An aqueous solution (2 ml) of cerium nitrate hexahydrate (0.43 g, 1 mmol*L*^-1^) was transferred to a glass tube, then a mixture of tetrabromoterephthalic acid (0.48 g, 1 mmol*L*^-1^), NaOH (0.08 g, 2 mmol*L*^-1^) and 4,4'-bpy (0.15 g, 1 mmol*L*^-1^) in ethanol/water (2 ml) was poured into the glass tube without mixing the two solutions. Colorless crystals began to form at ambient temperature during 1 month. One of these crystals was used for X-ray crystallography.

### Refinement

Hydrogen atoms bonded to carbon atoms, H1, H2, H3 and H4 were introduced at the positions calculated theoretically and refined using riding models with U_iso_(H) = 1.5 U_eq_(C). H5, H6, H7 are located in the Fourier difference maps but the positions of these atoms were not refined. Thermal parameteers have been fixed to 1.2 U_eq_(N) or 1.5 U_eq_(O), respectively.

### Crystal data

**Table:**

C_10_H_10_N_2_^2+^·C_8_Br_4_O_4_^2^^−^·2H_2_O	*Z* = 1
*M**_r_* = 673.93	*F*(000) = 324.00
Triclinic, *P*1	*D*_x_ = 2.161 Mg m^−^^3^
Hall symbol: -P 1	Mo *K*α radiation, λ = 0.71070 Å
*a* = 6.503 (3) Å	Cell parameters from 1137 reflections
*b* = 9.249 (4) Å	θ = 3.3–27.5°
*c* = 9.987 (4) Å	µ = 7.83 mm^−^^1^
α = 64.119 (14)°	*T* = 293 K
β = 85.868 (18)°	Prism, colorless
γ = 73.737 (14)°	0.30 × 0.20 × 0.20 mm
*V* = 517.9 (4) Å^3^	

### Data collection

**Table:**

Rigaku Mercury70 diffractometer	2116 reflections with *F*^2^ > 2.0σ(*F*^2^)
Detector resolution: 7.314 pixels mm^-1^	*R*_int_ = 0.027
ω scans	θ_max_ = 27.5°
Absorption correction: multi-scan (*REQAB*; Rigaku, 1998)	*h* = −8→8
*T*_min_ = 0.126, *T*_max_ = 0.209	*k* = −12→12
5065 measured reflections	*l* = −12→12
2336 independent reflections	

### Refinement

**Table:**

Refinement on *F*^2^	Secondary atom site location: difference Fourier map
*R*[*F*^2^ > 2σ(*F*^2^)] = 0.031	Hydrogen site location: inferred from neighbouring sites
*wR*(*F*^2^) = 0.086	H-atom parameters constrained
*S* = 1.19	*w* = 1/[σ^2^(*F*_o_^2^) + (0.0449*P*)^2^ + 0.1173*P*] where *P* = (*F*_o_^2^ + 2*F*_c_^2^)/3
2336 reflections	(Δ/σ)_max_ = 0.001
136 parameters	Δρ_max_ = 0.48 e Å^−^^3^
0 restraints	Δρ_min_ = −1.55 e Å^−^^3^
Primary atom site location: structure-invariant direct methods	

### Special details

**Table:**

Geometry. ENTER SPECIAL DETAILS OF THE MOLECULAR GEOMETRY
Refinement. Refinement was performed using all reflections. The weighted *R*-factor (*wR*) and goodness of fit (*S*) are based on *F*^2^. *R*-factor (gt) are based on *F*. The threshold expression of *F*^2^ > 2.0 σ(*F*^2^) is used only for calculating *R*-factor (gt).

### Fractional atomic coordinates and isotropic or equivalent isotropic displacement parameters (Å^2^)

**Table:**

	*x*	*y*	*z*	*U*_iso_*/*U*_eq_	
Br1	0.10645 (4)	0.42116 (4)	0.71999 (3)	0.03083 (11)	
Br2	0.32963 (5)	0.18104 (4)	0.54456 (3)	0.03350 (12)	
O1	0.1557 (4)	0.8335 (3)	0.5839 (3)	0.0351 (5)	
O2	0.3797 (4)	0.6584 (3)	0.7830 (3)	0.0422 (6)	
O3	0.7487 (4)	0.4074 (3)	0.9249 (3)	0.0446 (6)	
N1	0.8843 (4)	0.9226 (3)	0.7575 (3)	0.0306 (6)	
C1	0.8549 (6)	1.0592 (5)	0.7789 (4)	0.0409 (8)	
C2	0.7080 (5)	1.0919 (4)	0.8752 (4)	0.0368 (7)	
C3	0.5825 (4)	0.9836 (4)	0.9480 (3)	0.0236 (6)	
C4	0.6149 (5)	0.8444 (5)	0.9214 (4)	0.0367 (7)	
C5	0.7679 (6)	0.8159 (4)	0.8268 (4)	0.0377 (7)	
C6	0.4067 (4)	0.6003 (3)	0.5735 (3)	0.0221 (5)	
C7	0.3331 (4)	0.4652 (4)	0.5922 (3)	0.0229 (5)	
C8	0.4251 (4)	0.3656 (4)	0.5196 (3)	0.0231 (5)	
C9	0.3056 (5)	0.7067 (4)	0.6539 (3)	0.0262 (6)	
H1	0.9351	1.1338	0.7277	0.0491*	
H2	0.6927	1.1859	0.8916	0.0442*	
H3	0.5329	0.7698	0.9677	0.0441*	
H4	0.7906	0.7208	0.8110	0.0452*	
H5	0.9899	0.8937	0.6971	0.0367*	
H6	0.7539	0.3878	1.0277	0.0670*	
H7	0.6152	0.4970	0.8711	0.0670*	

### Atomic displacement parameters (Å^2^)

**Table:**

	*U*^11^	*U*^22^	*U*^33^	*U*^12^	*U*^13^	*U*^23^
Br1	0.02820 (17)	0.03494 (19)	0.03224 (19)	−0.01138 (13)	0.01655 (12)	−0.01780 (14)
Br2	0.03620 (19)	0.03384 (19)	0.0411 (2)	−0.01650 (14)	0.01468 (14)	−0.02352 (15)
O1	0.0340 (11)	0.0330 (11)	0.0321 (11)	0.0036 (9)	0.0083 (9)	−0.0172 (9)
O2	0.0477 (13)	0.0549 (14)	0.0277 (12)	−0.0052 (11)	0.0025 (10)	−0.0266 (11)
O3	0.0460 (14)	0.0483 (14)	0.0416 (14)	−0.0126 (11)	0.0168 (11)	−0.0237 (12)
N1	0.0286 (12)	0.0356 (13)	0.0320 (13)	−0.0080 (10)	0.0150 (10)	−0.0211 (11)
C1	0.0426 (18)	0.0430 (18)	0.050 (2)	−0.0230 (15)	0.0297 (16)	−0.0292 (17)
C2	0.0434 (17)	0.0342 (16)	0.0468 (19)	−0.0185 (14)	0.0247 (15)	−0.0287 (15)
C3	0.0239 (13)	0.0270 (13)	0.0226 (13)	−0.0063 (10)	0.0070 (11)	−0.0145 (11)
C4	0.0438 (17)	0.0400 (16)	0.0412 (18)	−0.0220 (14)	0.0226 (15)	−0.0276 (15)
C5	0.0479 (18)	0.0391 (17)	0.0413 (18)	−0.0177 (14)	0.0192 (15)	−0.0302 (15)
C6	0.0204 (12)	0.0252 (12)	0.0200 (13)	−0.0012 (10)	0.0033 (10)	−0.0127 (11)
C7	0.0197 (12)	0.0283 (13)	0.0208 (13)	−0.0048 (10)	0.0067 (10)	−0.0127 (11)
C8	0.0225 (12)	0.0226 (12)	0.0245 (13)	−0.0046 (10)	0.0043 (10)	−0.0121 (11)
C9	0.0277 (14)	0.0307 (14)	0.0274 (15)	−0.0097 (11)	0.0122 (11)	−0.0196 (12)

### Geometric parameters (Å, °)

**Table:**

Br1—C7	1.892 (3)	C6—C7	1.394 (5)
Br2—C8	1.887 (4)	C6—C8^ii^	1.396 (4)
O1—C9	1.248 (3)	C6—C9	1.516 (5)
O2—C9	1.249 (4)	C7—C8	1.395 (5)
N1—C1	1.332 (6)	O3—H6	0.963
N1—C5	1.331 (5)	O3—H7	1.002
C1—C2	1.374 (6)	N1—H5	0.941
C2—C3	1.392 (5)	C1—H1	0.930
C3—C3^i^	1.501 (4)	C2—H2	0.930
C3—C4	1.382 (6)	C4—H3	0.930
C4—C5	1.374 (5)	C5—H4	0.930
			
O1···H5^iii^	1.670	H5···O1^vi^	1.670
O1···H5^iv^	2.828	H5···O1^iv^	2.828
O2···H5^iii^	2.737	H5···O2^vi^	2.737
O2···H6^v^	1.944	H6···O2^v^	1.944
O2···H7	1.765	H7···O2	1.765
			
C1—N1—C5	120.5 (3)	C6^ii^—C8—C7	120.4 (3)
N1—C1—C2	121.2 (4)	O1—C9—O2	126.8 (4)
C1—C2—C3	119.6 (4)	O1—C9—C6	116.2 (3)
C2—C3—C3^i^	121.2 (4)	O2—C9—C6	116.9 (3)
C2—C3—C4	117.7 (3)	H6—O3—H7	110.0
C3^i^—C3—C4	121.1 (3)	C1—N1—H5	122.8
C3—C4—C5	120.1 (4)	C5—N1—H5	116.6
N1—C5—C4	120.9 (4)	N1—C1—H1	119.417
C7—C6—C8^ii^	118.8 (3)	C2—C1—H1	119.409
C7—C6—C9	120.1 (3)	C1—C2—H2	120.212
C8^ii^—C6—C9	121.1 (3)	C3—C2—H2	120.215
Br1—C7—C6	117.8 (3)	C3—C4—H3	119.938
Br1—C7—C8	121.4 (3)	C5—C4—H3	119.934
C6—C7—C8	120.8 (3)	N1—C5—H4	119.564
Br2—C8—C6^ii^	118.1 (3)	C4—C5—H4	119.572
Br2—C8—C7	121.6 (2)		
			
C1—N1—C5—C4	0.2 (5)	C8^ii^—C6—C7—C8	−0.0 (4)
C5—N1—C1—C2	1.5 (5)	C7—C6—C9—O1	−93.1 (3)
N1—C1—C2—C3	−2.2 (5)	C7—C6—C9—O2	86.7 (4)
C1—C2—C3—C3^i^	−178.9 (3)	C9—C6—C7—Br1	−0.1 (3)
C1—C2—C3—C4	1.1 (4)	C9—C6—C7—C8	−179.68 (19)
C2—C3—C3^i^—C4^i^	0.0 (4)	C8^ii^—C6—C9—O1	87.3 (4)
C2—C3—C4—C5	0.5 (4)	C8^ii^—C6—C9—O2	−93.0 (3)
C3^i^—C3—C4—C5	−179.5 (3)	C9—C6—C8^ii^—Br2^ii^	−0.8 (3)
C4—C3—C3^i^—C2^i^	−0.0 (4)	C9—C6—C8^ii^—C7^ii^	179.68 (19)
C3—C4—C5—N1	−1.2 (5)	Br1—C7—C8—Br2	−0.1 (3)
C7—C6—C8^ii^—Br2^ii^	179.51 (18)	Br1—C7—C8—C6^ii^	−179.58 (14)
C7—C6—C8^ii^—C7^ii^	0.0 (4)	C6—C7—C8—Br2	179.49 (18)
C8^ii^—C6—C7—Br1	179.59 (18)	C6—C7—C8—C6^ii^	0.0 (4)

Symmetry codes: (i) −*x*+1, −*y*+2, −*z*+2; (ii) −*x*+1, −*y*+1, −*z*+1; (iii) *x*−1, *y*, *z*; (iv) −*x*+1, −*y*+2, −*z*+1; (v) −*x*+1, −*y*+1, −*z*+2; (vi) *x*+1, *y*, *z*.

### Hydrogen-bond geometry (Å, °)

**Table:**

*D*—H···*A*	*D*—H	H···*A*	*D*···*A*	*D*—H···*A*
O3—H6···O2^v^	0.963	1.944	2.822 (4)	150.5
O3—H7···O2	1.002	1.765	2.766 (3)	177.2
N1—H5···O1^vi^	0.941	1.670	2.606 (4)	172.9

Symmetry codes: (v) −*x*+1, −*y*+1, −*z*+2; (vi) *x*+1, *y*, *z*.
